# Advisory Committee on Immunization Practices Recommended Immunization Schedule for Adults Aged 19 Years or Older — United States, 2020

**DOI:** 10.15585/mmwr.mm6905a4

**Published:** 2020-02-07

**Authors:** Mark S. Freedman, Paul Hunter, Kevin Ault, Andrew Kroger

**Affiliations:** ^1^Immunization Services Division, National Center for Immunization and Respiratory Diseases, CDC; ^2^University of Wisconsin, School of Medicine and Public Health, Madison, Wisconsin; ^3^City of Milwaukee Health Department, Milwaukee, Wisconsin; ^4^University of Kansas Medical Center, Kansas City, Kansas.

At its October 2019 meeting, the Advisory Committee on Immunization Practices (ACIP)* voted to recommend approval of the 2020 Recommended U.S. Adult Immunization Schedule for Persons Aged 19 Years and Older. The 2020 adult immunization schedule, available at https://www.cdc.gov/vaccines/schedules/index.html,^†^ summarizes ACIP recommendations in two tables and accompanying notes. This 2020 adult immunization schedule has been approved by the CDC Director, the American College of Physicians, the American Academy of Family Physicians, the American College of Obstetricians and Gynecologists, and the American College of Nurse-Midwives. Health care providers are advised to use the tables and the notes together.

ACIP’s recommendations on use of each vaccine are developed after in-depth reviews of vaccine-related data, including the epidemiology and burden of the vaccine-preventable disease, vaccine efficacy and effectiveness, vaccine safety, quality of evidence, feasibility of program implementation, and economic analyses of immunization policy (*1*). The adult immunization schedule is published annually to consolidate and summarize updates to ACIP recommendations on vaccination of adults and to assist health care providers in implementing current ACIP recommendations. The use of vaccine trade names in this report and in the adult immunization schedule is for identification purposes only and does not imply endorsement by ACIP or CDC.

For further guidance on the use of each vaccine, including contraindications and precautions, health care providers are referred to the respective ACIP vaccine recommendations at https://www.cdc.gov/vaccines/hcp/acip-recs/index.html. Changes in recommended use of vaccines can occur between annual updates to the adult immunization schedule. Information on these changes, if made, is available at https://www.cdc.gov/vaccines/acip/recommendations.html.^§^ Printable versions of the 2020 adult immunization schedule and ordering instructions are available at https://www.cdc.gov/vaccines/schedules/hcp/adult.html#note.

## Changes in the 2020 Adult Immunization Schedule

Changes in the 2020 adult immunization schedule for persons aged ≥19 years include new or revised recommendations for hepatitis A vaccine (HepA) (*2*); human papillomavirus vaccine (HPV) (*3*); influenza vaccine (*4*); serogroup B meningococcal vaccine (MenB); pneumococcal vaccine (*5*); and tetanus toxoid, reduced diphtheria toxoid, and acellular pertussis vaccine (Tdap) (*6*). Following are the changes to the cover page, Table 1, Table 2, and Notes.

**Cover page**

- Trademark symbols (®) were added to all vaccine trade names.
- PedvaxHIB was added to the table of trade names for *Haemophilus influenzae* type b vaccine.
- The footnote on the cover page has been edited and now reads “Do not restart or add doses to vaccine series if there are extended intervals between doses.”

**Table 1**

- **Age ranges:** The columns for age groups 19–21 years and 22–26 years have been combined, thereby reducing the number of columns for age ranges from five to four. This change was made because of the change in recommendation for catch-up HPV vaccination for all adults aged ≤26 years.
- **Tetanus, diphtheria, pertussis row:** This row has been edited to state that tetanus and diphtheria toxoids (Td) or Tdap may be used for the decennial tetanus booster.
- **Human papillomavirus (HPV) row:** The rows for males and females have been combined, reflecting that catch-up vaccination is now recommended for all adults aged ≤26 years. In addition, a blue box has been added for persons aged 27–45 years to indicate that shared clinical decision-making regarding vaccination is now recommended for this group.
- **Pneumococcal conjugate (PCV13) row:** The box for persons aged ≥65 years who do not have an additional risk factor or another indication has been changed to blue to indicate that shared clinical decision-making regarding vaccination is now recommended for this group.
- **Meningococcal B (MenB) row:** A blue box has been added for persons aged 19–23 years who are not at increased risk for meningococcal disease, indicating that shared clinical decision-making regarding vaccination is now recommended for this group.
- **Legend:** A blue box has been added to indicate that shared clinical decision-making is recommended regarding vaccination. The text defining the gray box has been edited and now reads “No recommendation/not applicable.”

**Table 2**

- **Tdap or Td row:** This row has been revised to read that Td or Tdap may be used for the decennial tetanus booster.
- **Human Papillomavirus (HPV) row:** This row has been combined into a single row including both males and females, reflecting that HPV vaccine is now recommended for all adults aged ≤26 years.
- **Hepatitis A (HepA) row:** The box for persons living with human immunodeficiency virus (HIV) infection (regardless of CD4 count) is now yellow, reflecting the new recommendation that previously unvaccinated persons in this group should be vaccinated.
- **Legend and bar text:** The gray box in the Legend has been edited and now reads “No recommendation/not applicable.” The red box has been edited and now reads “Not recommended/contraindicated — vaccine should not be administered.” The text appearing in the red bars has been changed from “Contraindicated” to “Not Recommended.”

**Notes**

- Edits have been made throughout the Notes section to harmonize language between the child/adolescent immunization schedule and the adult immunization schedule, where possible.
- A new subsection entitled “Shared Clinical Decision-Making” was added for each vaccine that includes this new ACIP recommendation (e.g., for HPV, PCV13, and MenB).
- **Hepatitis A:** The note was revised to include minor changes to the chronic liver disease definition, minor changes for the pregnancy indication, addition of the recommendation for vaccination in settings of exposure, and removal of clotting factor disorders as an indication for vaccination.
- **Hepatitis B:** The note was revised to include minor changes to the chronic liver disease definition and minor changes for the pregnancy indication.
- **Human papillomavirus:** The note was revised to indicate that HPV vaccination is recommended for all persons aged ≤26 years. A shared clinical decision-making subsection was added for persons aged 27–45 years.
- **Influenza:** The note was updated to include a bulleted list indicating when live attenuated influenza vaccine (LAIV) should not be used and minor edits to the guidance for persons with a history of Guillain-Barré syndrome.
- **Measles, mumps, and rubella:** The note was revised to clarify recommendations for health care personnel, with a separate bullet for personnel born in 1957 or later with no evidence of immunity and for health care personnel born before 1957 with no evidence of immunity.
- **Meningococcal:** The note was revised to include the use of the complement inhibitor ravulizumab as an indication for MenB administration in these patients. A shared clinical decision-making subsection was added that includes a bullet for adolescents and young adults aged 16–23 years who are not at increased risk for meningococcal disease. Under the “Special situations” section, the recommendation to administer a booster dose of MenB 1 year after the primary series and to revaccinate every 2–3 years if the risk remains was added.
- **Pneumococcal:** The note has been updated to reflect the updated recommendations for vaccination of immunocompetent (defined as adults without an immunocompromising condition, cerebrospinal fluid leak, or cochlear implants) adults aged ≥65 years. One dose of 23-valent pneumococcal polysaccharide vaccine (PPSV23) is still recommended. Shared clinical decision-making is recommended regarding administration of PCV13 to immunocompetent persons aged ≥65 years.
- **Tetanus, diphtheria, and pertussis:** The note has been updated to indicate that Td or Tdap may be used in situations where only Td vaccine was indicated for the decennial tetanus, diphtheria, and pertussis booster vaccination, tetanus prophylaxis for wound management, and catch-up vaccination.
- **Varicella:** The note has been updated to indicate that vaccination may be considered for persons with HIV infection without evidence of varicella immunity who have CD4 counts ≥200 cells/*μ*L.

## Additional Information

The Recommended Adult Immunization Schedule, United States, 2020 is available at https://www.cdc.gov/vaccines/schedules/hcp/adult.html and in the Annals of Internal Medicine (*7*). The full ACIP recommendations for each vaccine are also available at https://www.cdc.gov/vaccines/hcp/acip-recs/index.html. All vaccines identified in Tables 1 and 2 (except zoster vaccines) also appear in the Recommended Child and Adolescent Immunization Schedule for Ages 18 Years or Younger, United States, 2020.^¶^ The notes for vaccines that appear in both the adult immunization schedule and the child and adolescent immunization schedule have been harmonized to the greatest extent possible.

## Footnotes

* Recommendations for routine use of vaccines in adults are developed by Advisory Committee on Immunization Practices (ACIP), a federal advisory committee chartered to provide expert external advice and guidance to the CDC Director on use of vaccines and related agents for the control of vaccine-preventable diseases in the civilian population of the United States. Recommendations for routine use of vaccines in adults are harmonized to the greatest extent possible with recommendations made by the American Academy of Family Physicians (AAP) and the American College of Obstetricians and Gynecologists (ACOG). ACIP recommendations approved by the CDC Director become agency guidelines on the date published in the *Morbidity and Mortality Weekly Report* (*MMWR*). Additional information about ACIP is available at https://www.cdc.gov/vaccines/acip.

† Past immunization schedules are available at https://www.cdc.gov/vaccines/schedules/past.html.

§ CDC encourages organizations to use syndication as a more reliable method for displaying the most current and accurate immunization schedules on an organization's website rather than copying these schedules to their websites. Use of content syndication requires a one-time step that ensures an organization’s website displays current schedules as soon as they are published or revised; instructions for the syndication code are available on CDC’s website (https://www.cdc.gov/vaccines/schedules/syndicate.html). CDC also offers technical assistance for implementing this form of content syndication (e-mail request to ncirdwebteam@cdc.gov). Information on changes in ACIP recommendations in the adult immunization schedule before the next scheduled annual update, if any, is available at https://www.cdc.gov/vaccines/schedules/hcp/schedule-changes.html#adult.

¶ https://www.cdc.gov/vaccines/schedules/hcp/imz/child-adolescent.html.
